# Oral Health and Dental Care in Deaf and Hard of Hearing Population: A Scoping Review

**DOI:** 10.3290/j.ohpd.a44687

**Published:** 2020-07-04

**Authors:** Valeria Campos, Ricardo Cartes-Velásquez, Michael McKee

**Affiliations:** a Assistant Professor, Department of Preventive and Public Health Dentistry, School of Dentistry, University of Concepción, Concepción, Chile. Study concept and design, collected and interpreted the data, wrote and proofread the manuscript, contributed substantially to discussion.; b Associate Professor, Faculty of Dentistry, Universidad Andrés Bello, Concepción, Talcahuano, Chile. Study concept and design, interpreted the data, wrote and proofread the manuscript, contributed substantially to discussion.; c Assistant Professor, Department of Family Medicine, University of Michigan Medical School, Ann Arbor, MI, USA. Study design, interpreted the data, wrote and proofread the manuscript, contributed substantially to discussion.

**Keywords:** communication aids for disabled, deaf, hard of hearing, hearing loss, persons with disabilities, special care

## Abstract

**Purpose:**

To compile the literature available about the oral health and dental care of the deaf and hard of hearing (DHH) population.

**Materials and Methods:**

The study question of this scoping review was ‘What are the main findings reported in the literature about oral health and dental care of the DHH population?’ The following databases were included: Web of Science, LILACS, SciELO, MEDLINE, Scopus, EMBASE, GoogleScholar and Redalyc. Full-text articles published in peer-reviewed journals, in Spanish, Portuguese, and English, from the January 2000 to January 2018 were selected with qualitative and quantitative methods. All study designs were included in the review with the exception of letters to the editor and case reports

**Results:**

A total of fifty articles were selected for analysis. DHH population has poorer oral hygiene and a higher prevalence of caries than their non DHH peers. DHH also report significant struggles with oral health and dental access. Most dentists experienced difficulties communicating with their DHH patients

**Conclusions:**

This scoping review is the first known that centers on DHH oral health and their dental care. Efforts to develop accessible dental health programmes are needed to address apparent oral health inequities in the DHH population.

Hearing loss ranks third amongst disabilities.^104^ According to its aetiology, it can be genetic, congenital or acquired; according to its location, it can be classified as conductive, sensorineural, mixed, or central. Hearing loss can range from mild (less than 40 dB) to profound (more than 90 dB). Depending on the time of hearing loss onset, it can be prelingual, perilingual, or postlingual, which means before, during, or after language acquisition,^77,105^ or later onset (e.g. post-educational or post-vocational). Worldwide, more than 360 million people live with moderate to profound hearing loss. There is a prevalence of 1.4% in children between 5 and 14 years old, and about 10% among people 15 years old and above. A greater proportion of deaf and hard of hearing (DHH) live in low- and middle-income countries.^85,105^

People with prelingual hearing loss often identify themselves with the Deaf Community, a world with its own language and culture.^61^ The Deaf Community has several distinctive characteristics, customs and values which have emerged from their hearing condition. People in this community use sign language as their preferred method of communication.^37,43,105^ Sign language differs from spoken languages in its linguistic structure, is not universal,^43,87^ but is recognised in more than 30 countries.^26^

DHH individuals are more likely to have lower socioeconomic status, including lower household income, poorer educational achievement, and higher rates of unemployment.^17^ Furthermore, DHH individuals are associated with a number of adverse health outcomes.^34,99^ Hearing loss represents a major communication barrier in health care settings, impacting the quality of health care delivered to individuals with hearing loss.^13^ This is cause for concern, since poor health care communication adversely affects many health outcomes.^13,48,49^ Many DHH individuals struggle with lower health literacy and reading literacy level compared to non-DHH persons, ^14,47,94,68,100^ further complicating efforts to disseminate health information to this community. On the other hand, health personnel frequently lack training to address the specific needs of this population (e.g. communication, culture), which leads to patient dissatisfaction, reduced health care accessibility, inadequate information, and lower health care education and communication quality.^16,41,84^

Regular dental visits provide the basis of oral healthcare, so it is important that dentists obtain basic knowledge and competencies to deliver adequate dental care to the DHH population. Oral health is an important yet frequently overlooked element of a population’s health. Thus, little is known about the DHH oral health. In addition to the communication barriers experienced by the DHH, dentists similarly experience barriers to giving proper oral health care to this population. However, until now there no reviews have been available up to now which summarise the relevant issues on oral health and dental care in DHH.

The aim of this scoping review is to compile the literature about oral health and dental care of the DHH population.

## Materials and Methodology

### Research Question and Strategy

The guiding question of this scoping review was ‘What are the main findings reported in the literature about oral health and dental care of the DHH population?’ A DHH population was defined as those with hearing loss, either self-reported or objectively assessed by an audiometry instrument, in the primary articles included for this scoping review.

The search query included the following keywords: hearing impairment, deafness, deaf, hearing loss, people with disabilities, people with hearing impairment, oral health, dental care for persons with disabilities, Dental Plaque Index, DMF Index, Index of Orthodontic Treatment Need, Oral Hygiene Index, Periodontal Index, dentistry, dental health of people with disabilities, caries, oral hygiene, communication, bioethics, sign language, malocclusion, Deaf culture, Deaf community. The search query was adapted in an algorithm according to the requirements of each database.

As the literature used a range of criteria to categorise the DHH population, this situation was compensated by excluding articles regarding presbycusis (hearing loss asociated to aging) or with older DHH populations. However, most of the articles did not mention or clasiffied the severity/aetiology of the hearing loss, and the majority were children, giving more homogeonity to the studies.

A secondary search step included a ‘snowball’ technique to increase the number of relevant articles. For all articles found in the databases above, their literature citations were searched to find any other relevant articles that were not initially included. This process was repeated once more for articles included in the secondary step.

### Data Source

The databases were selected according to their coverage of biomedical disciplines: Web of Science, LILACS, SciELO, MEDLINE, Scopus, EMBASE, GoogleScholar and Redalyc. Initially, no limits were set on date, language, type of article, country, or any other filter.

### Eligibility Criteria

The following were included in the final analysis: full-text articles published in peer-reviewed journals; in Spanish, Portuguese, and English; from the January 2000 to January 2018. The exclusion criteria included letters to the editor and case reports; articles with no clear numeric results, using not validated indices or instruments, sample sizes less than 10 subjects, and combining results of the DHH population with other groups.

### Data Characterisation and Summary

For all articles, the following variables were recorded in an Excel spreadsheet: title, authors, country, year, type (qualitative/quantitative), methodological design, and comparisons with other populations.

For quantitative articles, the numeric data from indices or instruments used were gathered and summarised in tables and text. For qualitative articles, main topics and findings were summarised in the text.

## Results

### Characterisation of the Studies

A total of 51 articles were selected for analysis. Nineteen articles were published before and 32 were published after 2009; 15 were published in 2014 and 2015. The country of origin for most of the articles was India (17), followed by Brazil (6), Thailand (3), Romania (3), Nigeria (3), and Saudi Arabia (3). Three articles used qualitative methodology, and the remaining 48 used a quantitative methods. Among quantitative articles, 6 were experimental, and the remaining 42 were observational. Twenty studies included DHH and individuals with other disabilities; four studies included DHH and people without disabilities; and three included DHH, as well as people with other disabilities and those without disabilities.

### Preventive Dental Health

Multiple studies reported low rates of toothbrushing twice per day among DHH children and adolescents, ranging from 6%-14.7%.^60,67,72^ In China and Thailand, the rate of good oral hygiene among DHH was much lower than among non-DHH peers (e.g. toothbrushing 2x/day 15.3% vs 37.7%,^101^ and 86% vs 97%98). There appeared to be significant knowledge differences between non-DHH and DHH students on awareness of how to brush their teeth properly, with a prevalence of 79.1% and 55%, respectively.^101^

On the other hand, Suhani et al,^89^ using the WHO indices, reported a higher prevalence of deleterious oral habits, such as thumbsucking, mouth breathing, tongue thrust (53.3% vs 40.6%), as well as malocclusion (79.3% vs 57%) among DHH children compared to hearing children. Avasthi et al^12^ found a 59.78% prevalence of malocclusion signs, such as the presence of spacing, crowding, crossbite, increased overjet or others in DHH children.

Oral health educational interventions have demonstrated good results reducing gingival indices,^11,62^ bleeding,^11^ and plaque indices^7,11,62^ among DHH students. Furthermore, it has been found that a chlorhexidine gel containing aspartame or saccharin reduced the count of *Streptococcus mutans* in the deaf population.^29^

### DHH Oral Disease Burden

Caries prevalence in the DHH population varied widely between 18.18% and 95.7%, as shown on Table 1. Only three studies have made comparisons with non-DHH population. Chinese DHH adolescents have a caries prevalence of 55.9% and a DMFT index of 1.40 ± 1.89 vs 13.8% and 1.36 ± 1.72 in their non-DHH peers, respectively.^101^ In Brazil, DHH children have a caries prevalence of 46%, compared to 31% in non-DHH children.^42^ Also, in Thailand the prevalence of caries was 53.6% with a DMFT of 4.83 ± 4.39 and 50.6% and 3.90 ± 3.22 among DHH students and non-DHH, respectively.^98^

**Table 1:** DMFT, dmft and caries prevalence in DHH population

Country, year, reference	N	Age (years)	D	M	F	DMFT (SD)	d	m	f	dmft (SD)	Caries prevalence
Venezuela, 2003^44^	50	3–17	1.44	0.14	0.48	2.06	1.82	0.38	0.52	2.72	92%
Saudi Arabia, 2004^3^†	23	6–7	0.87	0	0	0.87 (1.25)	7.09	0.05	0.22	7.35 (3.82)	95.7%
	57	11–12	4.79	0.25	0.08	5.12 (3.45)	1.9	0.18	0.03	2.11 (2.53)	93%
India, 2008^39^	18	5–8	0.5	0	0 (0)	0.50 (0.79)	N/A	N/A	N/A	2.17 (1.98)	N/A
	37	9–12	1.81	0.02	0.02	1.76 (1.74)	N/A	N/A	N/A	1.59 (2.03)	93.33%
	43	13–17	2.67	0.12	0.16	2.95 (2.0)	N/A	N/A	N/A	0.16 (0.61)	87.4%
	29	18–22	3.48	0.62	0.38	4.48 (2.43)	N/A	N/A	N/A	0.00 (0.00)	N/A
India, 2014^74^	195	6–20	1.64	0.14	0.02	1.80 (1.26)	0.33	N/A	N/A	0.33 (0.24)	N/A
India, 2013^38^†	297	4–23	1.68	0.20	0.09	1.97 (1.93)	0.23	N/A	0.02	0.26 (0.85)	N/A
Iran, 2007^1^†	462	5–16	N/A	N/A	N/A	5.69	N/A	N/A	N/A	0.23	66%
Brazil, 2010^55^‡	50	3–12	N/A	N/A	N/A	N/A	N/A	N/A	N/A	N/A	46%
Thailand, 2014^98^‡	97	≥18	1.63	0.32	1.95	3.90 (3.22)	N/A	N/A	N/A	N/A	82.5%
India, 2015^52^†	132	3–15	0.74	0.02	0	0.76 (1.56)	0.73	0.01	0.03¥	0.77 (1.91)	N/A
Albania, 2014^33^†	147	3–18	N/A	N/A	N/A	4.7 (3.9)	N/A	N/A	N/A	2.8 (2.9)	88.4%*
											65.9%**
India, 2014^23^†	155	3–22	N/A	N/A	N/A	1.10 (1.58)	N/A	N/A	N/A	0.85 (1.76)	45.8%
India, 2013^66^†	95	7–17	1.38	0.02	0	1.4 (1.95)	0.34	0.14	0	0.47 (1.01)	N/A
China, 2012^101^‡	229	17–27	1.07	0.10	0.12	1.40 (1.89)	N/A	N/A	N/A	N/A	55.9%
South Africa, 2012^57^†	30	3–6	N/A	N/A	N/A	N/A	2.33	0.37	0.70	3.40 (3.87)	42.42%**
	33	7–9	N/A	N/A	N/A	N/A	1.73	1.06	0.18	2.97 (3.17)	
	13	10–12	0.15	0.08	0.00	0.23 (0.60)	N/A	N/A	N/A	N/A	18.18%*
	8	13–15	1.12	0.63	0.00	1.75 (3.24)	N/A	N/A	N/A	N/A	
	15	≥16	0.20	0.27	0.00	0.47 (0.92)	N/A	N/A	N/A	N/A	
India, 2016^72^	50	6–8	1.4	0.04	0.16	1.6 (1.3)	2.5	0.1	0.1	2.8 (2.2)	66%
	72	9–12	1.9	0.08	0.01	1.9 (1.2)	1.7	0.4	0.04	2.1 (1.5)	79.2%
	58	13–16	2.0	0.16	0.05	2.2 (1.2)	0.5	0.6	0.07	1.1 (1.3)	46.6%
India, 2005^10^	280	6–18	N/A	N/A	N/A	1.64	N/A	N/A	N/A	N/A	93.9%
India, 2014^80^†	200	5–16	N/A	N/A	N/A	2.1	N/A	N/A	N/A	1.3	69%
India, 2010^65^	137	7–18	2.46	1.20	0.00	2.53 (1.72)	N/A	N/A	N/A	N/A	35.32%
India, 2011^12^†	264	5–16	N/A	N/A	N/A	3.18	N/A	N/A	N/A	N/A	72.43%
Malaysia, 2015^63^	63	6–14	2.7	0.15	2.1	4.9 (3.28)	5.6	N/A	0.4	6.1 (4.14)	85%**
											88%*
Yemen, 2015^2^†	92	6–14	N/A	N/A	N/A	1.91 (2.07)	N/A	N/A	N/A	4.37 (3.11)	N/A
Kuwait, 2000^83^†	312	3–29	N/A	N/A	N/A	5.0	N/A	N/A	N/A	5.3	88.3%**
											83.6%*

* permanent dentition; ** deciduous dentition; † comparative study with other disabilities; ‡ comparative study with non-deaf population; ¥ filled, with caries

Additionally, from 65.3%^3^ to 79.5%^39^ of DHH needed single-surface restorations; according to Ajami et al,^1^ Mehta et al^53^ and Nqcobo et al,^58^ 100% of DHH subjects required dental treatment. Oredgduba et al^59^ and Reddy et al^66^ reported that from 88% to 100% of DHH subjects have never visited a dentist nor received dental care. According to Champion and Holt,^22^ 82 of 84 DHH children have visited a dentist, of whom 45 received dental care and 38 did not. In Thailand, 97.5% DHH had not received preventive dental care vs 84.2% of their non-DHH peers.^98^

Periodontal status and oral hygiene were evaluated using several indices across the studies included, as shown in Table 2.

**Table 2:** Periodontal and oral hygiene indices in the DHH population

Country, year, reference	N	Age (years)	index	Results (SD)				
India, 2008^41^	23	5–9	OHI–S	Mean score 1.57 (0.73).				
	48	10–14	OHI–S	Mean score 1.90 (0.67).				
	48	15–19	OHI–S	Mean score 1.88 (1.02).				
	8	20–24	OHI–S	Mean score 2.26 (0.94).				
	57	12–17	CPTIN	40% IPC0		42% IPC1		18% IPC2–4
	29	18–23	CPTIN	45% IPC0		24% IPC1		31% IPC2–4
India, 2005^64^	112	3–20	OHI–S	Mean score 1.49 (0.88).				
India, 2011^12^	264	5–16	Gingival index	Prevalence 9.65%				
India, 2010^65^	137	7–18	OHI–S	Mean score 1.49(0.76)				
			Löe and Sillness	Mean score 0.81(1.46)				
			OHI–S	Mean score 0.46 (0.31)				
			Attachment loss	0.26 (0.15)mm				
Albany, 2014^33^	147	3–18	OHI–S	Mean score 2.42.				
India, 2016^72^	180	6–16	CPTIN	Mean score 1.7 (0.61)				
India, 2013^38^	297	4–23	IPC	IPC_0_: 24.2%	IPC_1_: 21.2%	IPC_2_: 11.1%	IPC3: 35.4%	IPC4: 8.1%
India, 2014^73^	372	6–16	Löe and Sillness	Mean score 1.66				
			Gingivitis	Mean score 1.61				
India, 2012^7^	150	14–17	Plaque score	Mean score 1.25 (0.35)				
			OHI–S	Mean score 2.52 (1.08).				
			CPTIN	7.7% IPC_0_,				0.7% IPC_1_
India, 2015^61^	315	6 to >15	Plaque index*	Mean score 1.59.				
Thailand, 2012^11^	66	6–10	Löe and Sillness	Mean score 0.94.				
India, 2015^1^	56	5–17	Plaque index	Mean score 0.284 in permant teeth, 0.335 in mixed dentition, 0.437in deciduous teeth.				
Irán, 2007^74^	462	5–17	OHI–S	67.8% good		25.0% fair		8.2% poor
			Gingival index	51.1% good		39.6% fair		9.3% poor
Saudi Arabia, 2004^72^	23	6–7	Oral health index	17.4% good		60.9% fair		21.7% poor
	57	11–12	Oral health index	7% good		45.6% fair		47.4% poor
India, 2013^66^	95	7–17	OHI–S	Mean score 1.15 (0.72).				
Bulgaria, 2015^28^	100	5–12	OHI–S	Mean score 2.21 (0.54).				
India, 2015^52^	132	3–15	Gingival bleeding	Prevalence 66.6%				
Tanzania, 2008^79^	25	7–9	Gingival bleeding index	Mean score 0.13				
			Calculus index	Mean score 0.16.				
	51	10–12	Gingival bleeding index	Mean score 0.23				
			Calculus index	Mean score 0.32.				
	56	13–14	Gingival bleeding index	Mean score 0.29				
			Calculus index	Mean score 0.41				
	61	15–16	Gingival bleeding index	Mean score 0.36				
			Calculus index	Mean score 0.53				
	36	17–22	Gingival bleeding index	Mean score 0.46				
			Calculus index	Mean score 0.66				
Yemen, 2015^2^	92	6–14	Löe and Sillness	Mean score 1.19 (0.54)				
			Gingival index	Mean score 1.13 (0.60)				

*Turesky-Gilmore-Glickman with Quigley–Hein modification.

From 59.7% to 75% of DHH showed Angle class I occlusion,^1,25,59^ class II was found in 13% to 26%,^25,29,59^ and class III comprised between 8% and 10.8% in the DHH population.^1,25,59^

Using the Dental Aesthetics Index (DAI), it was reported that 77.1% of the DHH population have normal occlusion or slight malocclusion.^96^ According to the WHO indices, between 50.6%^96^ and 44.5%^31^ DHH subjects have normal occlusion, and 31.5%^31^ to 33.8%^78^ showed a slight malocclusion.

Using the Index of Orthodontic Treatment Need (IOTN), the Dental Health component did not differ statistically significantly between deaf and non-deaf teenagers (30% vs 22.4%).^5,6^ However, for the aesthetic component, the difference was statistically significant (43% vs 39.4%).^5^

### DHH Barriers to Dental Care

Qualitative studies found that DHH people rarely, if ever, can communicated effectively with their dentists.^20,30^ This demonstrates the need for communication provisions, including interpreters during healthcare encounters.^21,30^ Furthermore, parents of DHH children report always being the interpreter during dental sessions, even as the children age.^22^ This can compromise the children’s right to privacy. Parents of DHH children emphasise that dentists should be able to communicate directly and effectively with their children.^22^

DHH individuals report acceptable communication with their dentist only in very simple specific situations where complex explanations are not necessary (e.g. ‘spitting’). Furthermore, one article highlighted positive experiences from DHH patients when they received dental care from a DHH dentist who was able to effectively communicate with them, demonstrating the importance of cultural and communication competency.^21^

Regarding dental care, 46.15% DHH individuals self-reported experiencing discrimination, mainly because of communication issues, although further reasons are not reported.^33^ In addition, 87% of DHH^71^ and 61.1% of parents of DHH children^22^ reported communication barriers and/or breakdowns during their dental care. Furthermore, according to the findings of Rocha et al,^70^ only 22.3% of DHH people perceived good communication with the dentist.

The most commonly mentioned concerns when receiving dental care were: communication with the dentist (52.4% in a survey of parents of DHH children^22^ and 76%in a survey of adult DHH patients^71^), communication with the dental assistant (41.7%^22^ and 61.8%^71^), being called from the waiting room (38.1%^22^ and 68.1%^71^), understanding what will take place during the appointment (46.4%^22^ and 57.84%^71^), not pulling the face mask down to allow the DHH patient to lipread (32.9%^22^and 62%^71^) and the presence of background noise (36.5%^22^ and 55%^71^). 100% reported that dentists did not understand sign language.^32^ Such difficulties increased significantly with increased hearing loss severity.^71^

Regarding dental anxiety, Suhani et al^88^ found that 59.7% of DHH people have moderate to extreme dental anxiety, and 5.3% have dental phobia, which is statistically significantly more prevalent in people with previous negative experiences with dentists (48.4 ± 15.14 and 36.6 ± 17.8, respectively) (p < 0.001).

### Dentists’ Perceptions of DHH Dental Care

In two studies, most dentists experienced difficulties communicating with their DHH patients (97.5%^90^ and 56.2%^70^). Moreover, 68% of the dentists interviewed did not feel qualified to work with DHH patients.^90^ Dentists used a variety of communication methods with their DHH patients; 90.75% of dentists combined different methods, such as lipreading, writing, or sign language interpreters.^73^ According to Garbin et al,^33^ all of the dentists who cared for DHH individuals reported that family members or friends, not professional interpreters, functioned as their interpreters. The majority of dentists interviewed (60%) idenitfied costs as a reason for not hiring professional interpreters,^90^ and according to Rocha et al,^70^ 97.8% of the dentists reported the lack of an interpreter in their Family Health Care Units.

Regarding dental care, 69.7% of the dentists said that dental appointments with DHH patients required more time, while 34.5% felt that equitable dental care for DHH was not feasible.^90^ More worrying was the fact that one study demonstrated that 16% of dentists refused dental care provision to DHH patients due to their communication needs.^90^ However, this differed among DHH children. One study in Saudi Arabia found that 78% of dentists perceived that DHH children were able to receive the same orthodontic treatment as non-DHH children.^4^ Finally, 86.6% of dentists believed that DHH patients’ oral health was poorer than that of the general population.^90^

## Discussion

To the authors’ knowledge, this is the first scoping review focused on DHH oral health. This study demonstrates that DHH struggle with significant oral health and dental access difficulties. The findings of this study call for a systematic examination of the dental experiences, complications, costs, quality of care, and outcomes of DHH individuals. Given the cross-sectional nature of the articles included in the study, it is difficult to determine the causal factors associated with poor oral health in DHH populations.

The information available on oral health status of this population is limited. Although a great variability is apparent in the history of caries (DMFT/dmf), tooth decay (D/d) is the major contributor in all the articles cited, with the exception of Vichayarant et al,^98^ where the major contributor is restorations (F/f). In addition, the findings of Reddy et al^67^ and Oregduba et al,^60^ where over 80% of the DHH population have never visited a dentist, clearly expresses the need for dental treatment in this population. Regarding malocclusions and periodontal health, there are not enough comparative studies with a non-DHH population to establish an association; in addition, general agreement of the indices used is lacking, which makes it difficult to make accurate comparisons between these studies. Finally, although DHH populations largely experience poorer oral hygeine, there are successful examples in which this can be reversed through appropriate oral health education through visual methods.^11,62,73^

There are several potential factors that may contribute to the above disparities. First, hearing loss represents a major source of miscommunication in the health care setting.^51^ This affects a variety of health-related outcomes, especially health knowledge, behaviour, treatment adherence, and patient satisfaction.^15,27,52,86,93^

Second, the DHH struggle with lower health literacy and access to health information, including incidental learning opportunities.^48^ poor health literacy affects the quality of health care, including oral health,^57^ and may result in poorer oral health outcomes for DHH individuals. Also, multiple studies demonstrated lower health knowledge among DHH individuals on a variety of medical topics.^36,50,57,63,91,102,103^It not known whether this is the same for oral health knowledge among DHH individuals.

Third, DHH individuals are more likely to be poorer and require public assistance, including public dental insurance. Blanchfield et al^17^ analysed data from multiple national datasets (NHIS, NHANES, NHISD) and found that DHH individuals were significantly more likely to be publicly insured, unemployed, and have lower family incomes. Lower socioeconomic status has been shown to be a strong driver of decreased access to dental health care.^35^

DHH people communicate via sign language, speech, lipreading or a combination thereof. Regardless the communication method, many of the above articles point out the importance of good health care provider awareness on how to effectively communicate with DHH patients in clinical settings.^55^ Specifically, for patients with limited English proficiency, the use of professional language interpreters in the UK is correlated with improved clinical care, and DHH report positive experiences in health care encounters when experienced professional sign language is offered. Also, in New Zealand, DHH access to professional interpreters is associated with advantages such as access to better health services and more information, improved ability to engage in leisure activities and live in a healthy environment.^41^ On the other hand, in Brazil, it was concluded that speech is not sufficient to establish a link between the DHH patient and the health professional.^24^ It is important to point out that as most of the DHH populations included in this study are functionally illiterate because of the educational and social barriers they face every day, written notes and speech are not adequate for communication.^24,33,82,84,95^

The lack of availability of specialised oral health personnel for DHH individuals in primary care is due to the absence of training in their curricula, and this scenario is repeated in every other health profession. It was found that health professionals are not adequately prepared to care for DHH patients.^19,24^ In addition, a high proportion of health professionals do not feel qualified to meet the needs of the DHH; they report a lower probability of providing health care to the DHH and point out that these patients are the most complex to address due to communication barriers.^40,53,70,90^ This situation hinders the delivery of health services, putting at risk the successful treatment of these patients.

Due to the many communication barriers and existing oral health disparities, dentists and oral health professionals should consider strategies to address these gaps. This may include training on how to effectively communicate with DHH patients, establishing relationships with professional interpreters, and providing accessible oral health programmes to increase knowledge on good oral hygeine and techniques. It is necessary to understand the daily reality to which DHH patients are exposed, in order to create a health professional-patient connection, improve trust and patient satisfaction, increase patients’ use of preventive health measures and health appointment attendance, thus benefitting their health.^76^ There are very few but still successful programmes that have been developed for the training of medical, dental and pharmacy students in treating DHH patients, resulting in professionals with better attitudes towards the DHH. Yet these programmes have not been established as a mandatory part of the curricula.^41,44,45-47, 69,40,75,76,92,97^

A promising effort in Chile, through the funding from the National Disability Service, has developed approaches aimed at eliminating the communication issues DHH experience in dental care, involving the joint work of dentists, deaf people and Chilean sign language interpreters using mobile software that gives relevant information about dental care. This facilitates diagnosis and treatment, and improves the oral health care experience of the deaf patient through pre-recorded sign language videos.^18^ Other efforts around the globe have proved successful in addressing DHH health inequities. For example, the establishment of specialised primary health-care centres, although scarce, has been well received by DHH patients, since they aim to eliminate the communication, health education and access barriers previously described. In Scotland, the ‘Sensory Support Centre WISC’ has a high level of health professional-patient satisfaction with the service received; handicapped patients particularly appreciate the way in which WISC staff demonstrate knowledge and empathy with the challenges they face due to sensory impairment (visual or hearing loss), improving their quality of life.^83^ In Austria, the ‘Health Centre for the Deaf’ has been developed where true health access is provided through staff competent in deaf culture who are able to communicate in sign language. DHH patients also have access to education programmes on diabetes and to mental health care.^41^ Finally, in France, there is an outpatient service for the primary care of DHH people, which has also been favourably received by the population.^9^

### Limitations

The study limitations warrant consideration. First, there was a relative paucity of good quality publications and data on DHH oral health. The articles varied in the data type, methodology, and DHH sample (e.g. deaf signers vs individuals with any hearing loss).

Studies on DHH dental health use several indices, especially for periodontal disease; thus is quite difficult to determine the magnitude of oral diseases in this population. Also, there is a geographically asymetric distribution of the studies, as most of them are from Asia, with limited studies in the Americas. This conveys unrepresentative data from DHH populations around the globe. This is noteworthy, as there are countries were health access for DHH population is greater, meaning that this population probably enjoys better dental health.

Moreover, only four studies compared the DHH population’s oral health with a hearing population. This lack of comparative studies hinders the posibility of assessing a clear impact of hearing disabilities on dental health.

Despite these limitations, this review helps to demonstrate areas of oral health needs for the DHH population.

## Conclusion

This scoping review is the first to focus on DHH persons’ oral health and their dental care, which is a neglected issue in oral health reseach worldwide. The review highlights the need for further research using longitudinal data and standarised measures of oral health to understand the causes of oral health disparities in the DHH population. In this regard, special considerations must be taken, as the approach to the DHH population demands social and communication adjustments. Tailored health programmes are needed to educate DHH on how to adequately care for their teeth. Thus, efforts to develop accessible dental health programmes are needed to address apparent oral health inequities in the DHH population.
